# The immune checkpoint adenosine 2A receptor is associated with aggressive clinical outcomes and reflects an immunosuppressive tumor microenvironment in human breast cancer

**DOI:** 10.3389/fimmu.2023.1201632

**Published:** 2023-09-11

**Authors:** Basma Zohair, Dounia Chraa, Ibtissam Rezouki, Hamza Benthami, Ibtissam Razzouki, Mohamed Elkarroumi, Daniel Olive, Mehdi Karkouri, Abdallah Badou

**Affiliations:** ^1^Immuno-Genetics and Human Pathology Laboratory (LIGEP), Faculty of Medicine and Pharmacy, Hassan II University, Casablanca, Morocco; ^2^Team Immunity and Cancer, The Cancer Research Center of Marseille (CRCM), Inserm, 41068, CNRS, UMR7258, Paoli-Calmettes Institute, Aix-Marseille University, UM 105, Marseille, France; ^3^Department of Pathological Anatomy, Ibn Rochd University Hospital Center, Casablanca, Morocco; ^4^Mohamed VI Oncology Center, Ibn Rochd University Hospital Center, Casablanca, Morocco; ^5^Mohammed VI Center for Research & Innovation, Rabat, Morocco and Mohammed VI University of Sciences and Health, Casablanca, Morocco

**Keywords:** A2AR, PD-1, CTLA-4, tumor and immune microenvironment, immunosuppression, immune checkpoint, immunotherapy, breast cancer prognosis

## Abstract

**Background:**

The crosstalk between the immune system and cancer cells has aroused considerable interest over the past decades. To escape immune surveillance cancer cells evolve various strategies orchestrating tumor microenvironment. The discovery of the inhibitory immune checkpoints was a major breakthrough due to their crucial contribution to immune evasion. The A2AR receptor represents one of the most essential pathways within the TME. It is involved in several processes such as hypoxia, tumor progression, and chemoresistance. However, its clinical and immunological significance in human breast cancer remains elusive.

**Methods:**

The mRNA expression and protein analysis were performed by RT-qPCR and immunohistochemistry. The log-rank (Mantel-Cox) test was used to estimate Kaplan-Meier analysis for overall survival. Using large-scale microarray data (METABRIC), digital cytometry was conducted to estimate cell abundance. Analysis was performed using RStudio software (7.8 + 2023.03.0) with EPIC, CIBERSORT, and ImmuneCellAI algorithms. Tumor purity, stromal and immune scores were calculated using the ESTIMATE computational method. Finally, analysis of gene set enrichment (GSEA) and the TISCH2 scRNA-seq database were carried out.

**Results:**

Gene and protein analysis showed that A2AR was overexpressed in breast tumors and was significantly associated with high grade, elevated Ki-67, aggressive molecular and histological subtypes, as well as poor survival. On tumor infiltrating immune cells, A2AR was found to correlate positively with PD-1 and negatively with CTLA-4. On the other hand, our findings disclosed more profuse infiltration of protumoral cells such as M0 and M2 macrophages, Tregs, endothelial and exhausted CD8+ T cells within A2ARhigh tumors. According to the Single-Cell database, A2AR is expressed in malignant, stromal and immune cells. Moreover, it is related to tumor purity, stromal and immune scores. Our results also revealed that CD8+T cells from A2ARhigh patients exhibited an exhausted functional profile. Finally, GSEA analysis highlighted the association of A2AR with biological mechanisms involved in tumor escape and progression.

**Conclusion:**

The present study is the first to elucidate the clinical and immunological relevance of A2AR in breast cancer patients. In light of these findings, A2AR could be deemed a promising therapeutic target to overcome immune evasion prevailing within the TME of breast cancer patients.

## 1 Introduction

Despite considerable progress in cancer management, breast cancer remains a major public health concern given its high morbidity and mortality rate, with an estimated 2.3 million new cases and 685,000 deaths worldwide in 2020 (1, 2). Breast cancer accurately reflects intratumoral heterogeneity conditioning therapeutic strategy. While chemotherapy remains the backbone of treatment for triple-negative breast cancer (TNBC), endocrine and human epidermal growth factor receptor 2 (HER2) targeted therapies provide the gold standard for hormone receptor-positive (HR+) and HER2-positive (HER2+) tumors, respectively (3, 4). In addition to TNBC and HER2+ tumors’ propensity for recurrence, early metastasis, and poor survival, patients harboring these stubborn tumors are prone to build-up conventional therapy resistance (3–15). Although chemotherapy is widely perceived as the mainstay of TNBC treatment, this therapeutic approach reflects a detrimental aspect with some clinical drawbacks. One of the adverse effects of chemotherapy involves growth promotion and activity of cancer cell intravasation niches, called tumor microenvironment of metastasis (TMEM), which endows the tumor with aggressive features and dramatically affects the clinical outcome of patients (16, 17). The success of immunotherapy in patients with immune-sensitive tumors has brought this treatment strategy to the forefront of current oncology breakthroughs (18–20). Therefore, immune checkpoint inhibitors (ICIs), notably anti-PD-1 and anti-CTLA-4 mAbs have received widespread interest over the past decade. However, despite the clinical benefit of ICIs in some tumor contexts, these have not been proven to be highly effective in TNBC and HER2+ patients (5, 18–22). Indeed, tumors appear to be able to overcome effects of ICIs through various strategies, including synergistic engagement of several immunosuppressive pathways (23). Interestingly, recent studies have reported compensatory upregulation of inhibitory immune checkpoints in patients receiving ICI therapy (24–26). Among these regulatory molecules, A2AR represents one of the most prominent and essential pathways in the TME. Known as a member of the G protein-coupled receptor (GPCR) family, this adenosine (ADO) receptor is expressed on nearly all immune cells (27).

As is the case with most solid tumors, 25% to 40% of invasive breast carcinomas are hallmarked by hypoxic areas driving extracellular ATP release with an overexpression of hypoxia-inducible factor-1 alpha (HIF-1α) (27, 28). The latter serves as a potent enhancer of CD39 and CD73 ectonucleotidase expression, which in turn mediate ATP, ADP, and AMP hydrolysis and consequently extracellular ADO accumulation (27, 29–31). Under physiological conditions, A2AR signaling upholds immune homeostasis to safeguard tissues against the onset of autoimmune disorder (32, 33). Nevertheless, in the cancer setting, the stimulation of this receptor via its ligand ADO triggers signal transduction of cAMP/PKA/CREB pathway while damping that of NFκB and JAK/STAT to inhibit the antitumor function of immune cells (27, 34). Thus, A2AR impairs the proliferative potential, effector and cytotoxic activity, as well as CD8+T cell infiltration within the TME (35–38). The attenuation of A2AR-mediated TCR and CD28 signaling drives CD8+T cells into an exhausted state marked by altered production of IFNγ, PRF and GZMB with upregulation of inhibitory immune checkpoints including PD-1, CTLA-4, LAG-3 and TIM-3 (27, 39–41). A2AR engagement also acts by preventing the maturation, proliferation and cytotoxicity of NK cells, while impairing the neoantigen presentation ability of dendritic cells (DC) (38, 42, 43). Otherwise, the A2AR pathway strengthens the immunosuppressive behavior of protumoral immune cells by hindering macrophage-induced phagocytosis, improving myeloid-derived suppressor cells (MDSC) function and promoting Tregs and M2-like macrophage polarization (38, 44–46). The A2AR receptor may also impinge on the non-immune axis of the TME, inducing tumor growth, epithelial-mesenchymal transition (EMT), and angiogenesis, thereby contributing to metastasis (36, 47–51).

Gastric, colorectal, and renal carcinomas have provided evidence of the link and involvement of A2AR in the poor prognosis of cancer patients (47, 48, 52, 53). Genetic and pharmacological inhibition of this immunosuppressive pathway has shown significant efficacy reflected by tumor burden decrease and metastasis prevention in experimental models (36, 54, 55). In renal cell carcinoma, phase I results from the first clinical trial of A2AR antagonist exhibited durable clinical improvement with immune response restoration even in patients resistant or refractory to PD-1/PD-L1 inhibitors (56). Given the complexity and heterogeneity of breast tumors and the large proportion of non-responders to currently available ICIs, the aim of the present study was to investigate the clinical and immunological relevance of A2AR in human breast cancer.

## 2 Materials and methods

### 2.1 Patients and specimen collection

**Figure 1:**
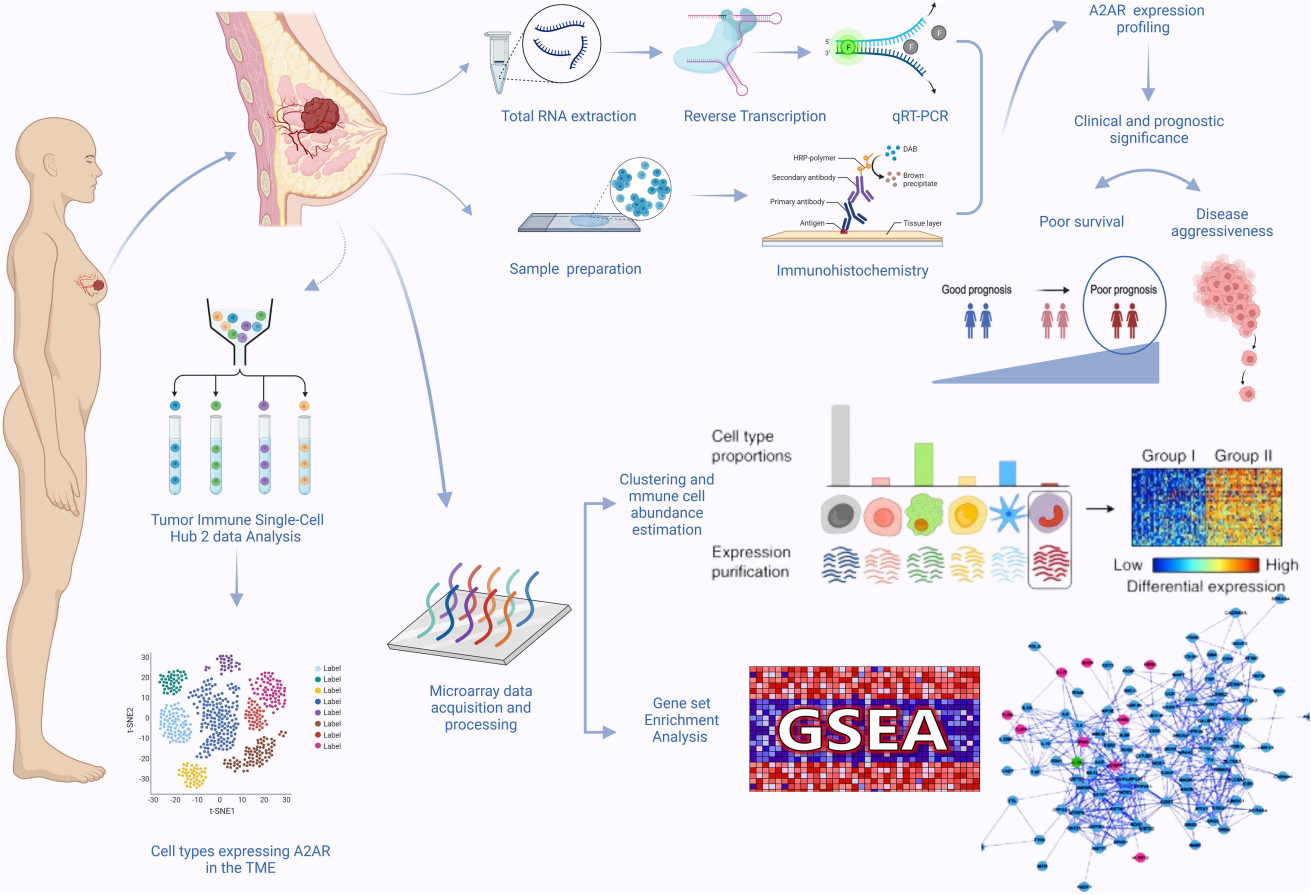
Graphical abstract.

Our study workflow is illustrated in (**Figure 1**). The present study includes 62 patients with invasive breast carcinoma who underwent surgical treatment between 2018 and 2021. The age of patients ranged from 32 to 89 years, with an average of 51 years. A total of 124 fresh specimens consisting of tumor tissues (n = 62) and matched adjacent tissues (n = 62) from the same patients were collected immediately after surgical resection at the Mohamed VI Oncology Center, Ibn Rochd University Hospital Center, Casablanca, Morocco. Tissue samples harvested from the uninvaded area adjacent to the tumor served as a control. Estrogen receptor (ER), Progesterone receptor (PR) and HER2 status were determined by the pathologists according to the American Society of Clinical Oncology/College of American Pathologists (ASCO/CAP) guidelines. Scarff-Bloom-Richardson (SBR) grading and histological subtyping were evaluated following standard recommendations.

Eligible patients were selected based on the following criteria: patients diagnosed with invasive breast carcinoma who underwent mastectomy or conservative surgery, free and informed consent, available formalin-fixed, paraffin-embedded tissue blocks and patients with defined molecular subtypes (Luminal A, Luminal B, HER2+ or TNBC). However, the exclusion criteria include male patients, unavailability of free and informed consent, unavailability of matched control tissue and incomplete medical records.

### 2.2 METABRIC dataset acquisition and preprocessing

Transcriptomic and clinicopathological data of 1904 primary invasive breast carcinoma tumors were collected from the large-scale METABRIC (Molecular Taxonomy of Breast Cancer International Consortium) cohort. For this purpose, we exported (METABRIC, Nature 2012 & Nat communication 2016) dataset using the cBioPortal for Cancer Genomics interface (https://www.cbioportal.org/). Clinicopathological parameters included in data_clinical_patient.txt and data_clinical_sample.txt files were merged and mapped to the corresponding gene expression data. The transcriptome file comprises mRNA expression levels of 24,368 genes measured by the Illumina Human v3 microarray, log2 transformed and normalized. To predict the 10-year survival rate, Nottingham Prognostic Index (NPI) scores were converted and categorized into 4 prognostic groups: Excellent, Good, Moderate and Poor.

Only patients with complete transcriptomic data were included in this study. In contrast, male patients or those with incomplete data were excluded. All analyses were repeated several times independently by two investigators.

### 2.3 Total RNA extraction, reverse transcription and quantitative real-time PCR

Total RNA was extracted from 124 fresh biopsies (breast carcinoma and matched control tissue) using TRIzol reagent (Invitrogen, France), according to the manufacturer’s instructions. After the estimation of total RNA concentration and quality by a NanoVueTM Plus spectrophotometer (GE Healthcare, UK), cDNA was synthesized from 0.5 µg of RNA included in a reaction mixture containing RNase-Free Water and Random Hexamer Primer (Bioline, France) and incubated at 70°C for 5 min. Afterward, Tetro reverse transcriptase buffer, RNase-free water, RNase inhibitor (Invitrogen, France), dNTP (10 mM), and Tetro reverse transcriptase enzyme (Bioline, France) were added, followed by incubation at 25°C for 10 min, then at 45°C for 30 min, and finally at 85°C for 5 min.

Real-time PCR was performed using SYBR Green PCR Master Mix (Thermo Fischer) on the Bio-Rad CFX96 Real-Time PCR System. Specific primer pairs targeting each gene were used at 10 μM concentration. All experiments were carried out according to the following schedule: holding stage at 95°C for 10 min, followed by 40 cycles of denaturation at 95°C for 15 s, then annealing and extension at 60°C for 1 min. The specificity control of PCR reaction was applied after each experiment by analyzing the amplicon melting curves. A second specificity-checking was implemented by submitting the PCR product (the amplified cDNA) to agarose gel electrophoresis. Data were assessed as a relative mRNA expression using the housekeeping gene ß-actin and matched control tissue as internal controls. The relative quantification was computed using the 2^-ΔΔCt^ approach. Only the comparative analysis of tumor and matched control tissues was conducted by applying the 2^-ΔCt^ method.

Primer pairs used in this study:

**Table:** 

Gene	Forward sequence	Reverse sequence
*β-actin*	5′- GAGATGGCCACGGCTGCTT-3′	5′- GCCACAGGACTCCATGCCCA-3′
*ADORA2A*	5’-ATCGCCATTGACCGCTACAT3-’	5’-GCTGACCGCAGTTGTTCCA-3’

### 2.4 Immunohistochemistry

Formalin-fixed, paraffin-embedded (FFPE) specimens from 45 invasive breast carcinoma and 10 matched control tissues were sectioned at an optimal thickness of 3-4 µm. Histologic sections were oven-dried at 60°C for one hour and then left at 37°C overnight prior to any treatment. The sections were then deparaffinized and rehydrated prior to heat-induced epitope unmasking using the PT Link system (Dako, Denmark). This antigen retrieval step was performed with a (low or high pH) solution providing a 3-in-1 pretreatment (EnVision Flex target retrieval solution low/high PH (× 50), Dako, Denmark). Samples were incubated with peroxidase-blocking reagent (EnVision flex peroxidase-blocking reagent, Dako, Denmark) for 5 min at room temperature and then rinsed with wash buffer (EnVision flex wash buffer, Dako, Denmark).

Thereafter, sections were incubated with the primary antibodies (A2AR clone 7F6-G5-A2 (Santa Cruz Biotechnology Inc.) at a 1:50 dilution, PD-1 clone DBM15.5 (Diagnostic BioSystems) at a 1:100 dilution and CTLA-4 clone F-8 (Santa Cruz Biotechnology Inc.) at a 1:500 dilution for 1 hour at room temperature. Negative control sections were incubated with Isotype control antibodies (Mouse IgG2a Isotype Control clone PPV-04 (OriGene) at a 1:500 dilution and Mouse IgG1 Isotype Control clone MOPC-21 (LSBio) at a 1:200 dilution for each sample. Otherwise, different positive control tissues were added for each primary antibody used. After washing, the secondary antibody (EnVision Flex/HRP, Dako, USA) was added and slides were incubated for 20 min at room temperature. The latter were then rinsed and incubated with a DAB substrate-chromogen solution (EnVision DAB+chromogen, Dako, USA) for 10 min.

Subsequently, slides were immersed in a hematoxylin bath for counterstaining and dehydrated in 3 ethanol baths (70%, 96%, and 100%). Finally, they were cleared in toluene baths and then mounted for reading under an Olympus light microscope (Olympus, Tokyo, Japan).

### 2.5 Immunostaining assessment and scoring system

Staining intensity, localization (membrane, cytoplasm, or nucleus), and percentage of labeled tumor, immune, and endothelial cells were evaluated by two independent pathologists. For gene expression analysis, a semi-quantitative assessment of immunostaining, presented as a Histoscore (H-score), was performed. This approach combines the intensity of staining and the percentage of labeled cells. Staining intensity was considered as negative (0), weak (1), intermediate (2) or strong (3). The H-score was calculated as follows: (1 x % of weak positive cells) + (2 x % of moderate positive cells) + (3 x % of strong positive cells). Thus, the expression level was ranged from 0 to 300.

### 2.6 Computational analysis of tumor-infiltrating immune cells

To assess the abundance of tumor infiltrating immune cells and to estimate tumor purity, stromal and immune scores, the computational deconvolution approach was performed using RStudio software version (7.8 + 2023.03.0) and four algorithms based on different immunological signatures: EPIC, CIBERSORT, ImmuneCellAI, and ESTIMATE. Prior to processing, the METABRIC transcriptomic dataset was standardized and converted into a non-log linear matrix. Then, according to A2AR gene expression and using the median as the cutoff, we stratified our cohort into two patient groups (A2AR^low^ and A2AR^high^).

### 2.7 Gene Set Enrichment Analysis (GSEA)

To investigate the key signaling pathways and biological processes linked to A2AR, we performed Gene Set Enrichment Analysis using RStudio software version (2023.03.0) and exploiting the three molecular signature databases: Hallmark, Curated and Ontology gene sets. Enriched terms with a false discovery rate (FDR) and a (p-nominal) < 0.05 are considered statistically significant.

### 2.8 A2AR exploration at single-cell resolution

The scRNA-seq Tumor Immune Single-cell Hub 2 (TISCH2) database is used to investigate the distribution of A2AR expression in different cell populations. The cell type annotation of three breast cancer datasets: BRCA_EMTAB8107, BRCA_GSE114727_10X and BRCA_Alex was arranged in two levels: Malignancy and Major Lineage. The manifold learning algorithm (UMAP) is adopted for dimension reduction. A2AR expression is explored in malignant, stromal and immune cells.

### 2.9 Statistical analysis

Statistical analysis, graphical representations and Heat map visualization were performedusing GraphPad Prism 8.0.1, RStudio software version 7.8, Morpheus (Broad Institute) and BioRender. For Overall survival, Kaplan–Meier analysis was estimated using the Log-rank (Mantel-Cox) test. To determine A2AR gene expression status, the median is used as a cutoff to stratify our METABRIC and experimental cohorts into A2AR^low^ and A2AR^high^ clusters. The non-parametric two-sided Wilcoxon signed rank test was applied for matched-pairs analysis. The Mann-Whitney rank test was conducted for unpaired analysis. Correlation coefficients were estimated with Pearson’s r statistic. Analysis with a 2-sided P value less than 0.05 (p < 0.05) was considered statistically significant.

### 2.10 Study approval

All experiments were conducted in conformity with the principles set forth in the Helsinki declaration and approved by the Ethics Committee for Biomedical Research (CERB) of Ibn Rochd University Hospital Center, under the approval code (28/15). The free and informed consent form was signed by all subjects participating in this study. Medical records containing clinical and pathological data (age, stage, grade and histological and molecular subtypes) were obtained from the hospital pathology department.

METABRIC patients are anonymous and their data are publicly available. The authors of the original publication have obtained free informed consent from all participants (57), therefore, this part of the present study was exempt from Institutional Review Board approval requirements.

## 3 Results

### 3.1 Human breast tumor exhibit increased levels of A2AR compared to matched uninvaded control tissue

**Figure 2:**
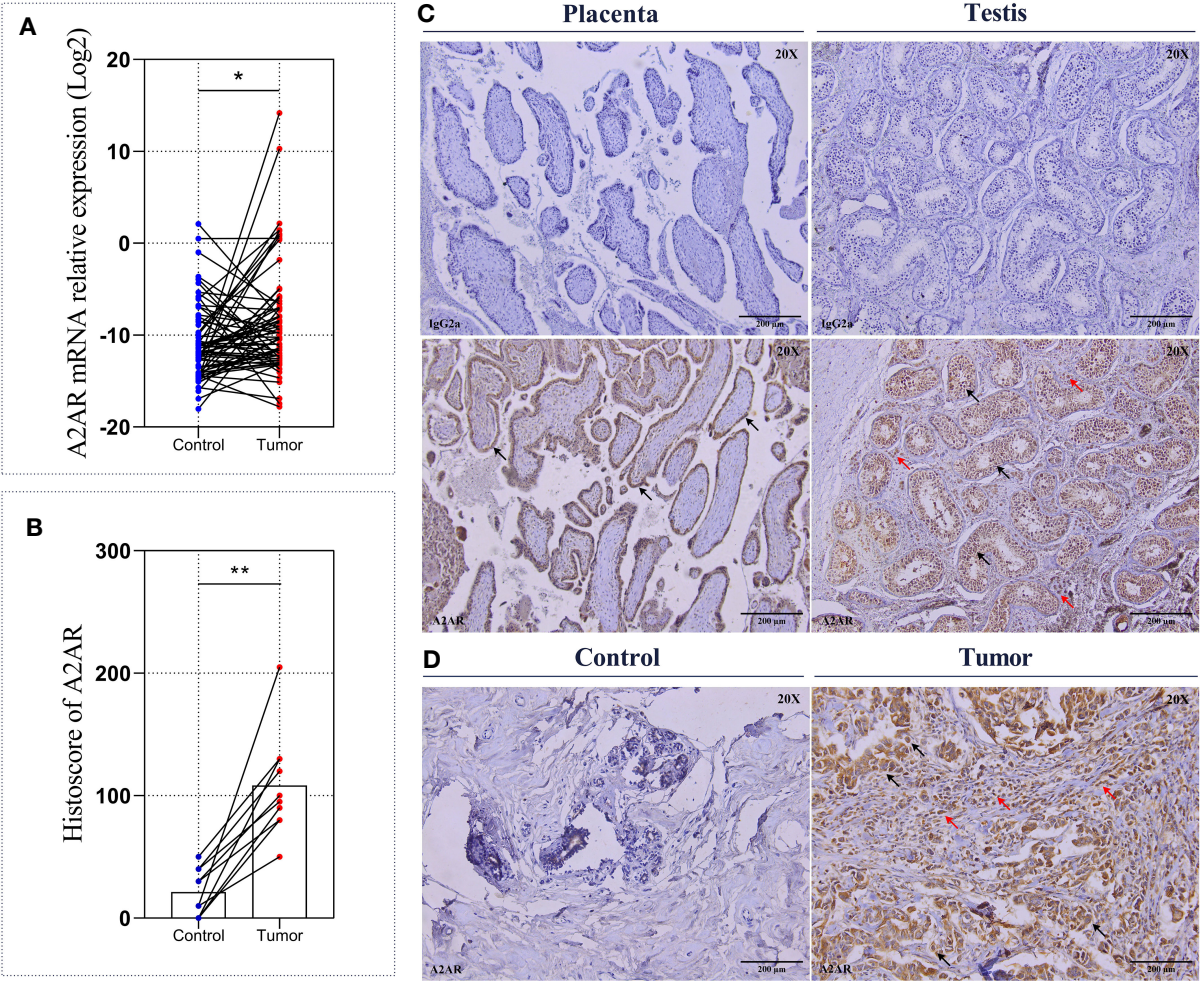
A2AR expression on breast tumors and matched control tissues. The A2AR expression level was measured by qRT-PCR and immunohistochemistry. **(A)** A2AR gene expression exhibits an elevated level in breast tumors compared to matched control tissues (*p* = 0.0176). **(C)** Representative immunohistochemical staining for A2AR and the IgG2a isotype (magnification20X, scale bar 200µm) in positive control tissues: Placenta (black arrows indicate tubular epithelial lining cells) and Testis (black arrows indicate germline cells at different development stages, and red arrows show Leydig cells). **(D)** A2AR staining showed membrane and cytoplasmic localization within both tumor and immune cells (black arrows indicate tumor cells, and red arrows show immune cells). **(B, D)** A2AR protein expression is more pronounced within tumors compared to matched control tissues (*p* = 0.0020). Significance was calculated using the Wilcoxon matched-pairs signed rank test. *p<0.05, **p<0.01.

In order to highlight the clinical impact of A2AR and determine its eventual involvement in human breast tumorigenesis, a cohort of 62 invasive breast carcinoma patients with an average age of 51 years (ranging from 32 to 89 years) was included in this study. The mRNA relative expression of *ADORA2A* gene, encoding human A2AR was assessed by qRT-PCR in 124 fresh specimens. Comparative analysis of 62 tumor tissues and 62 matched control tissues revealed increased expression of A2AR in breast tumors (**Figure 2A**). To corroborate these findings, we evaluated A2AR expression at the protein level by performing immunohistochemical staining in tumor and matched control tissues from 10 patients. The IgG2a Isotype was used as a negative control, while the placenta and testis were included as positive control tissues (**Figure 2C**). Immunological labeling revealed membrane and cytoplasmic expression of A2AR protein in both immune and cancer cells (**Figure 2D**). Interestingly, quantification of A2AR H-score for each sample exhibited higher expression within the tumor compared to matched uninvaded control tissue (**Figures 2B, D**). These findings suggest that A2AR might potentially contribute to the pathogenesis of human breast cancer.

### 3.2 A2AR is associated with aggressive clinical features and predicts poor overall survival in breast cancer patients

**Figure 3:**
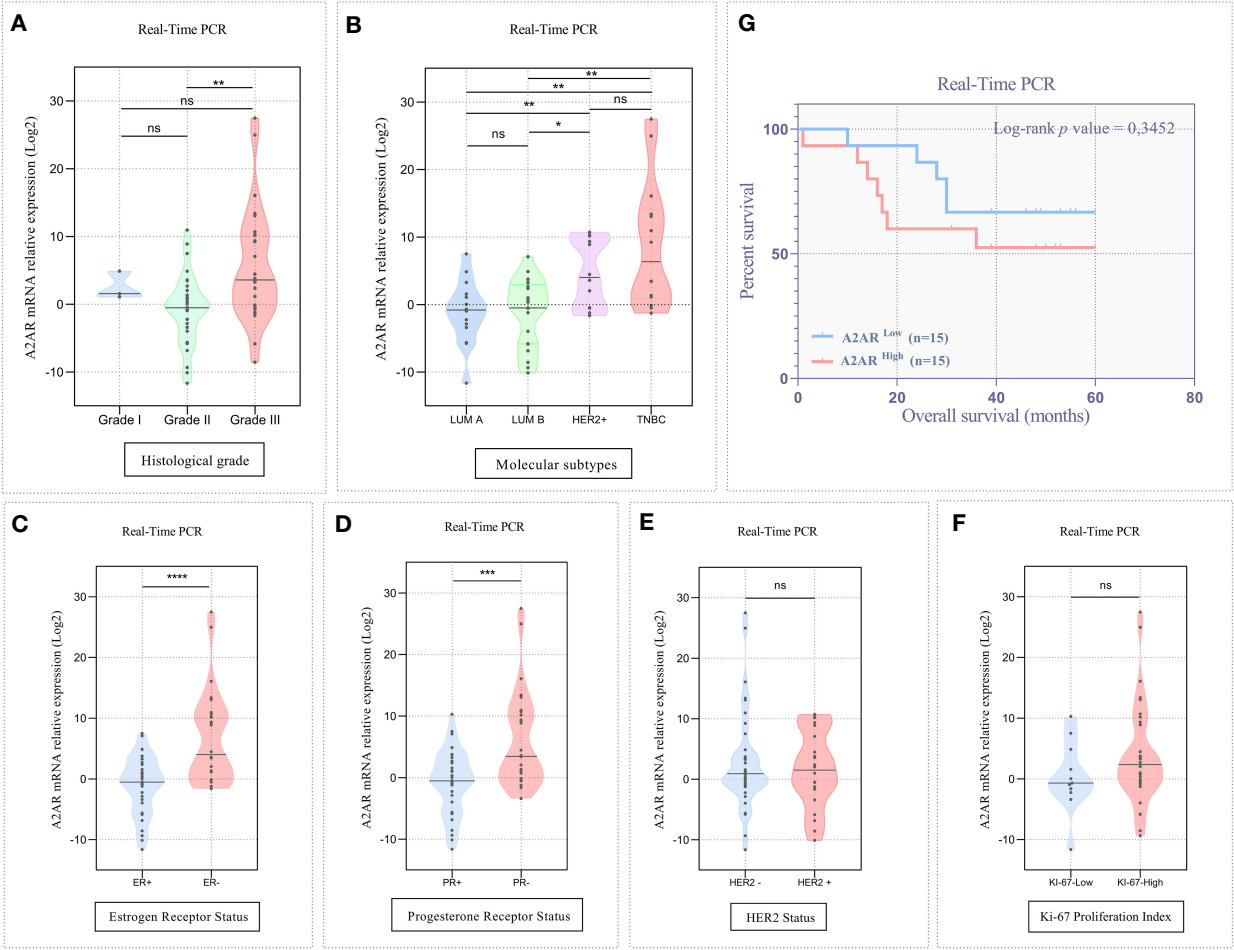
A2AR transcript level is linked to unfavorable clinicopathological outcomes. **(A, B)** The A2AR mRNA relative expression is significantly increased in high grade (grade II vs grade III: *p* = 0.0019), HER2+ (HER2+ vs LumA: *p* = 0.0087), (HER2+ vs LumB: *p* = 0.0162) and TNBC tumors (TNBC vs LumA: *p* = 0.0011), (TNBC vs LumB: *p* = 0.0018). **(C, D)** A2AR gene expression is strongly elevated in tumors with ER- (*p* < 0,0001), and PR- (*p* = 0,0007) status. **(E, F)** A2AR has no association with HER2 (*p* = 0.9388) status and KI-67 proliferation index (*p* = 0.2130). **(G)** Kaplan–Meier analysis reveals that A2AR gene expression is not related to survival (*p* = 0.3452). Significance was calculated using the Mann-Whitney and the Log-rank (Mantel-Cox) tests. *p<0.05, **p<0.01, ***p<0.001, ****p<0.0001, ns, not significant.

Given the increased levels of A2AR within the mammary tumor, we aimed to explore its clinical value for our patients by investigating its association to well-established breast cancer prognostic features. The clinicopathological parameters of patients are summarized in (**Table 1**). In high-grade tumors (grade III), an overexpression of A2AR was detected by the transcriptional analysis (**Figure 3A**). Our findings further revealed an association with the most aggressive molecular subtypes, known for their poor prognosis, by showing a significant upregulation of our gene of interest in TNBC and HER2+ patients (**Figure 3B**). Estrogen and progesterone receptors and human epidermal growth factor status constitute independent risk factors which affect prognosis and predict response to immunotherapy. Consequently, the transcript-level study illustrated the association between A2AR and hormone receptor status with unfavorable prognosis (ER- and PR-) (**Figures 3C, D**). In contrast, analysis of HER2 status (**Figure 3E**) showed no significant difference in expression between groups. Ki-67 is another distinct parameter considered for decades as a prognostic marker related to disease aggressiveness (58). In order to evaluate A2AR expression according to the tumor proliferation index, we stratified our cohort into two groups, Ki-67^low^ (≤20%) and Ki-67^high^ (>20%). However, although Ki-67^high^ tumors seem to exhibit a strong A2AR transcript level trend (**Figure 3F**), the difference is not statistically significant.

**Table 1:** Clinicopathological parameters of the experimental cohort.

Clinicopathological parameters	Real-Time PCR		Immunohistochemistry	
	No.	(%)	No.	(%)
Histological grade Grade I Grade II Grade III	3 31 28	4.84 50.00 45.16	4 17 24	8.89 37.78 53.33
Molecular subtypes Luminal A Luminal B HER2+ TNBC	15 21 12 14	24.19 33.87 19.36 22.58	11 14 10 10	24.45 31.11 22.22 22.22
Estrogen receptor status (ER) ER+ ER-	36 26	58.06 41.94	24 21	53.33 46.67
Progesterone receptor status (PR) PR+ PR-	35 27	56.45 43.55	24 21	53.33 46.67
HER2 status HER2- HER2+	40 22	64.52 35.48	31 14	68.89 31.11
Ki-67 proliferation index Ki-67 Low Ki-67 High	11 34	24.44 75.56	14 31	31.11 68.89

HER-2, human epidermal growth factor receptor-2; TNBC, triple negative breast cancer; ER, estrogen receptor; PR, progesterone receptor.

**Figure 4:**
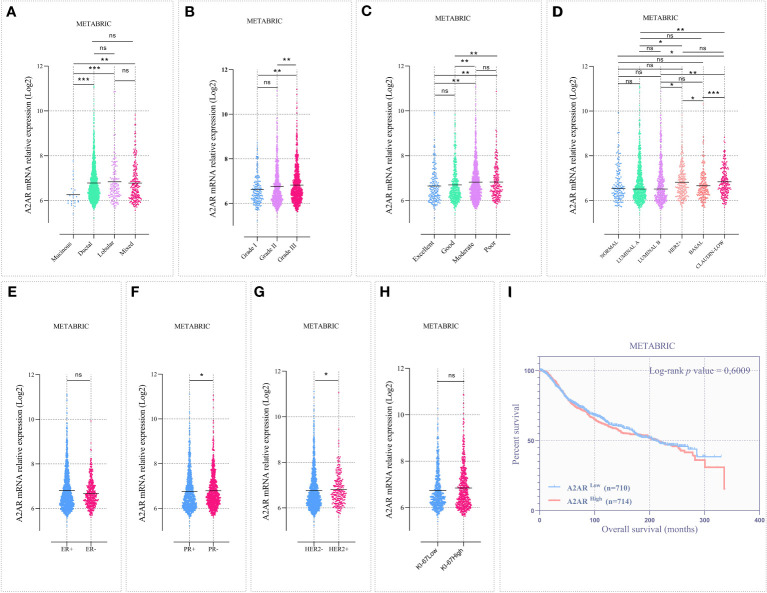
The A2AR gene expression is associated with aggressive clinical features in the METABRIC cohort. Microarray data from 1904 patients with invasive breast carcinoma were analyzed. **(A)** A2AR expression is downregulated in mucinous subtype tumors compared to ductal (*p* = 0.0002), lobular (*p* = 0.0005) and mixed (*p* = 0.0015). **(B)** A2AR is overexpressed in high-grade tumors compared to grade I (*p* = 0.0027) and grade II (*p* = 0.0064). **(C)** Patients presenting poor (poor vs excellent: *p* = 0.0067), (poor vs good: *p* = 0.0048) or moderate (moderate vs excellent: *p* = 0.0071), (moderate vs good: *p* = 0.0011) prognostic index exhibit high levels of A2AR transcripts. **(D)** Tumors with an aggressive subtype such as HER2+ (HER2+ vs. LumA: *p* = 0.0265), (HER2+ vs. LumB: *p* = 0.0204) and Claudin Low (Claudin Low vs. Normal: *p* = 0.0113), (Claudin Low vs. LumA: *p* = 0.0012), (Claudin Low vs. LumB: *p* = 0.0017) show increased A2AR expression. **(F, G)** A2AR gene level is linked to PR- (*p* = 0.0359) and HER2+ (*p* = 0.0160) status. **(E, H)** A2AR shows no association with ER (*p* = 0.6840) and Ki-67 (*p* = 0.0601) status. **(I)** Kaplan–Meier analysis reveals that A2AR gene expression is not related to survival (*p* = 0.6009). Significance was calculated using the Mann-Whitney and the Log-rank (Mantel-Cox) tests. *p<0.05, **p<0.01, ***p<0.001, ns, not significant.

The large-scale METABRIC dataset was also investigated to support the transcriptomic findings from our cohort. To this end, microarray expression data from 1904 patients with primary invasive breast carcinoma were explored. Patient clinicalpathological parameters are described in (**Supplementary Table 1**). Analysis of public data showed that A2AR is linked to ductal, lobular and mixed histological subtypes (**Figure 4A**). In accordance with the experimental cohort, High-grade tumors displayed increased A2AR expression (**Figure 4B**). As illustrated in (**Figure 4D**), the molecular subtyping of the METABRIC dataset included two additional subgroups (Normal and Claudin-low). In addition to its adverse prognosis, the latter represents a distinctly aggressive subgroup, related to stemness characteristics, downregulation of major cell junction components and activation of the EMT process during tumor progression (59, 60). Interestingly, our data showed the association of A2AR with Claudin-low and HER2+ subtypes. Furthermore, A2AR mRNA levels was increased in patients exhibiting PR- and HER2+ status (**Figures 4F, G**), however, no significant difference was detected between groups of ER status and Ki-67 proliferation index (**Figures 4E, H**).

Although the management of breast cancer is mainly based on well-defined clinical features, this pathology is characterized by an extremely complex and heterogeneous molecular profile. Therefore, the NPI was established to predict the clinical outcome of patients (prediction of 10-year survival after surgery). This prognostic index is widely used in clinical practice and has undergone prospective validation after long-term follow-up in large multicentric studies. The NPI is computed by combining three histopathological criteria (grade and size of tumor and lymph node invasion). Consequently, we performed the NPI analysis by stratifying the cohort into 4 prognostic groups. Thus, we showed that A2AR was linked to patients with moderate to poor survival prediction (**Figure 4C**).

**Figure 5:**
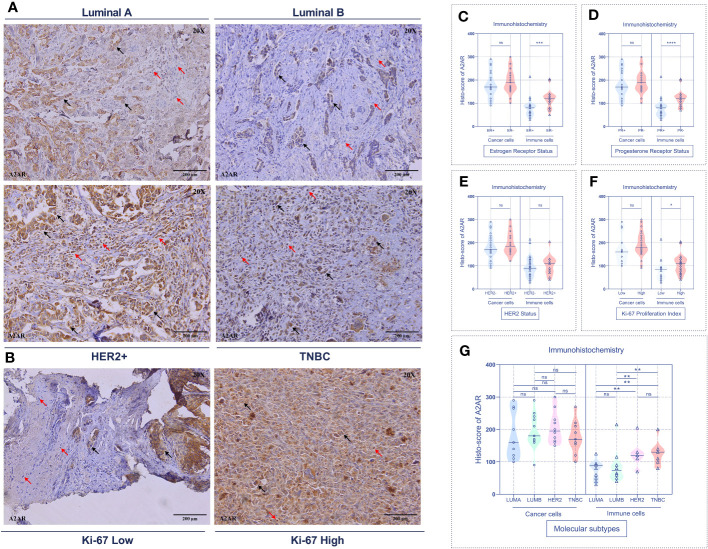
A2AR protein is associated with aggressive molecular subtypes and a high proliferation index. **(A, B)** Representative immunohistochemical staining (magnification 20X, scale bar 200µm) showing A2AR expression according to molecular subtypes and Ki-67 proliferation index status. **(C–F)** A2AR is overexpressed in immune cells from tumors with status: ER- (p = 0.0003), PR- (p < 0.0001) and high Ki-67 proliferation index (p = 0.0473). **(G)** A2AR is highly expressed in immune cells of HER2+ (HER2+ vs. LumA: p = 0.0073), (HER2+ vs. LumB: p = 0.0054) and TNBC (TNBC vs. LumA: p = 0.0032), (TNBC vs. LumB: p = 0.0035) tumors. Significance was calculated using the Mann-Whitney test. Black arrows indicate tumor cells. Red arrows show immune cells. *p<0.05, **p<0.01, ***p<0.001, ****p<0.0001, ns, not significant.

**Figure 6:**
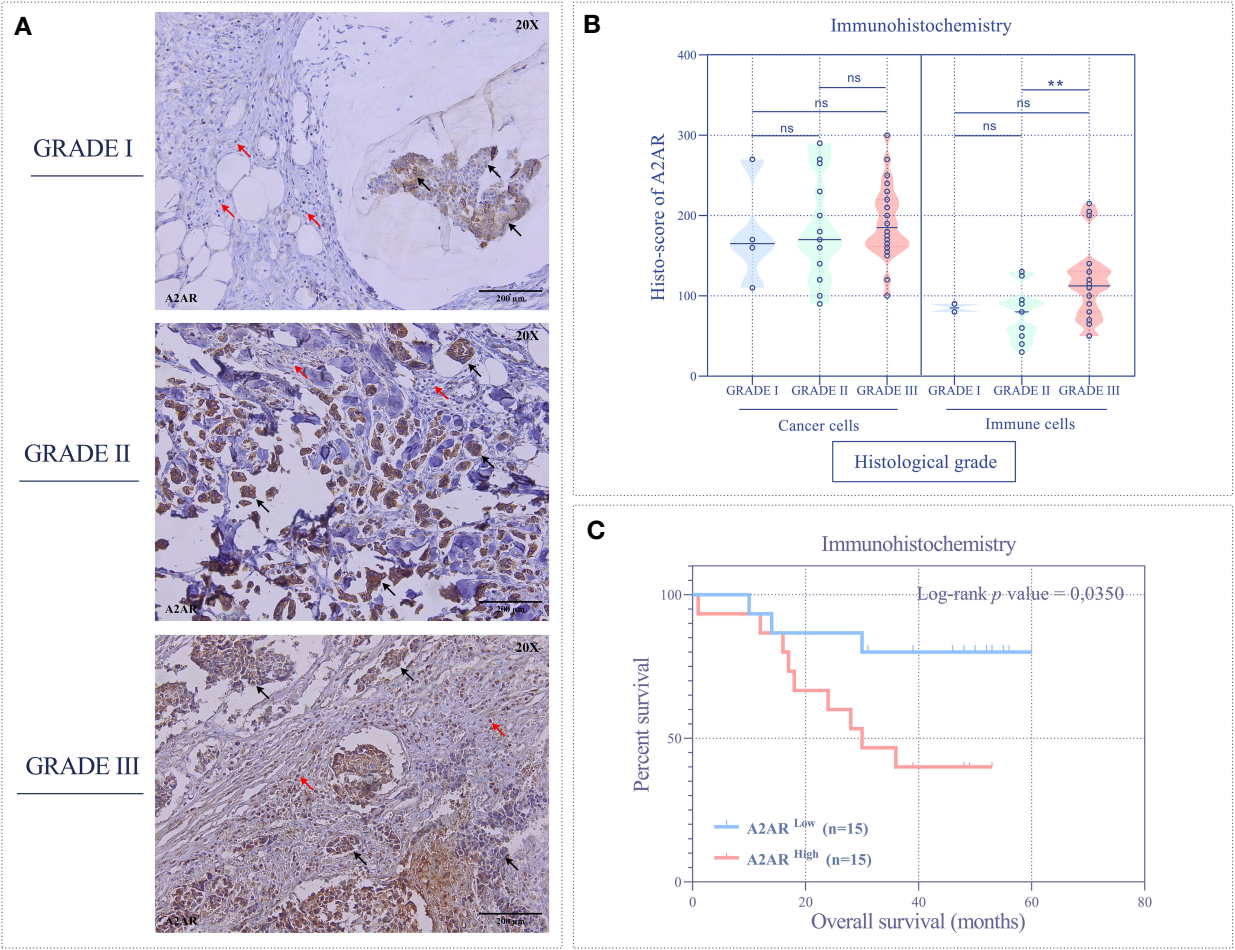
The A2AR protein is associated with high grade and predicts poor survival. **(A)** Representative immunohistochemical staining (magnification 20X, scale bar 200µm) of A2AR according to different histological grades. **(B)** A2AR shows high expression in immune cells from high-grade tumors (grade III) compared to those from grade II (*p* = 0.0054). **(C)** Patients overexpressing A2AR (A2AR^high^) predict poor overall survival (*p* = 0.0350). Significance was calculated using the Mann-Whitney and the Log-rank (Mantel-Cox) tests. Black arrows indicate tumor cells. Red arrows show immune cells. **p<0.01, ns, not significant.

To substantiate these findings, we further analyzed the expression of our molecule of interest at the protein level by immunohistochemistry. Immunological staining was performed on tumor specimens from 45 patients. For each sample, H-score of cancer cells and tumor-infiltrating immune cells were estimated independently. Consistent with the transcriptomic data, A2AR expression on tumor-infiltrating immune cells was significantly associated with ER- and PR- status (**Figures 5C, D**), HER2+ and TNBC molecular subtypes (**Figures 5A, G**), as well as high tumor grade (**Figures 6A, B**). However, A2AR was not associated with HER2 status (**Figure 5E**). Furthermore, in contrast to the transcriptomic data, immunohistochemical staining revealed increased levels of A2AR protein in Ki-67^high^ tumors (**Figures 5B, F**). This discrepancy between gene and protein expression profiles could be ascribed to an eventual post-transcriptional regulation. Surprisingly, the analysis of tumor cells did not show any association between A2AR and clinicopathological parameters.

Finally, we evaluated the prognostic value of A2AR by estimating overall survival. Accordingly, patients were stratified into two groups, A2AR^low^ and A2AR^high^. Clustering was performed according to A2AR expression using the median as a cutoff. At the transcriptomic level, Kaplan-Meier analysis estimated by the Log-rank (Mantel-Cox) test showed no significant difference between groups in the experimental (**Figure 3G**) and METABRIC (**Figure 4I**) cohorts. Interestingly, at the protein level, survival curves reflect the association of A2AR with a worse prognosis. In fact, A2AR^high^ patients exhibit poor overall survival compared to the A2AR^low^ group (**Figure 6C**). Therefore, our findings illustrate the prognostic impact of A2AR expression by predicting adverse clinical outcomes and negatively affecting the overall survival of breast cancer patients. In this regard, it should be emphasized that A2AR might be involved in breast cancer progression and aggressiveness mainly through the immunological process.

### 3.3 A2AR is remarkably correlated with PD-1 and CTLA-4 inhibitory immune checkpoints

**Figure 7:**
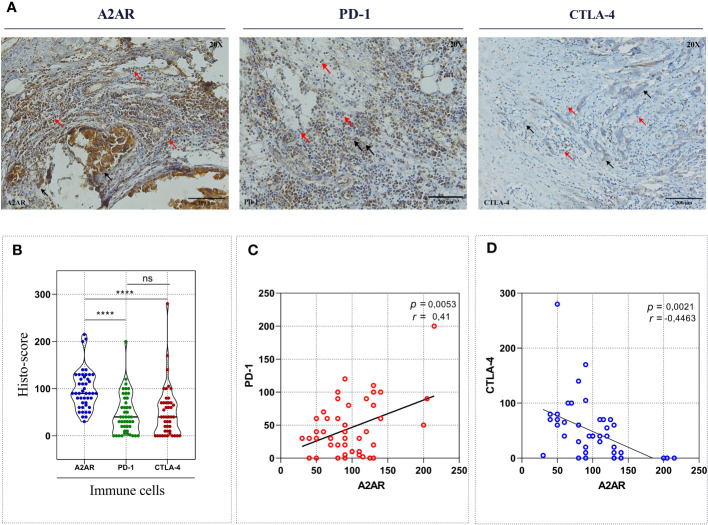
A2AR exhibits a significant correlation with PD-1 and CTLA-4 inhibitory immune checkpoint molecules. **(A)** Representative Immunohistochemical staining of A2AR, PD-1 and CTLA-4 (magnification 20X, scale bar 200µm). **(B)** A2AR protein seems to have the strongest expression compared to PD-1 (*p* < 0.0001) and CTLA-4 (*p* < 0.0001). **(C, D)** The expression of A2AR correlated positively with PD-1 (*p* = 0.0053, r = 0.41) and negatively with CTLA-4 (*p* = 0.0021, r = -0.44). Statistical difference was calculated using the Wilcoxon matched-pairs signed rank test. Pearson’s rank coefficient was used for correlation. Black arrows indicate tumor cells. Red arrows show immune cells. ****p<0.0001, ns, not significant.

Admittedly, in some tumor contexts, most notably melanoma, ICIs have proved to be considerably effective by achieving more durable antitumor responses than conventional therapies. Nevertheless, they have not been successful in breast cancer management, particularly for HER2+ and TNBC cancers, which are defined as immunogenic tumors. Indeed, only a restricted subset of metastatic TNBC is responsive to these immunotherapeutic agents with an overall response rate reaching 10%. Several studies have provided compelling evidence for the involvement of compensatory and synergistic immune checkpoint mechanisms in ICI monotherapy resistance. In this regard, we aimed to investigate the correlation of A2AR with PD-1 and CTLA-4 regulatory proteins to identify the potential interplay between these immunological pathways and consequently emphasize the relevance of combined therapy in human breast cancer. As a first result, our immunohistochemical analysis revealed that among these three regulators, A2AR protein exhibit the strongest expression in human breast tumor infiltrating immune cells (**Figures 7A, B**). Subsequently, Pearson’s coefficient showed a positive correlation between A2AR and PD-1 protein (**Figure 7C**). However, as depicted in (**Figure 7D**), our protein of interest displays a negative correlation with CTLA-4. Taken together, these findings imply that the prevailing immunosuppression within the mammary TME may be more related to the immunosuppressive effect of A2AR and an eventual interplays with PD-1 and CTLA-4 checkpoints might exist. Therefore, we suggest that precision immunotherapy management in breast cancer requires a careful focus on the status of different immunological biomarker expression.

### 3.4 A2AR is closely linked to the biological processes underlying tumorigenesis and breast cancer progression

**Figure 8:**
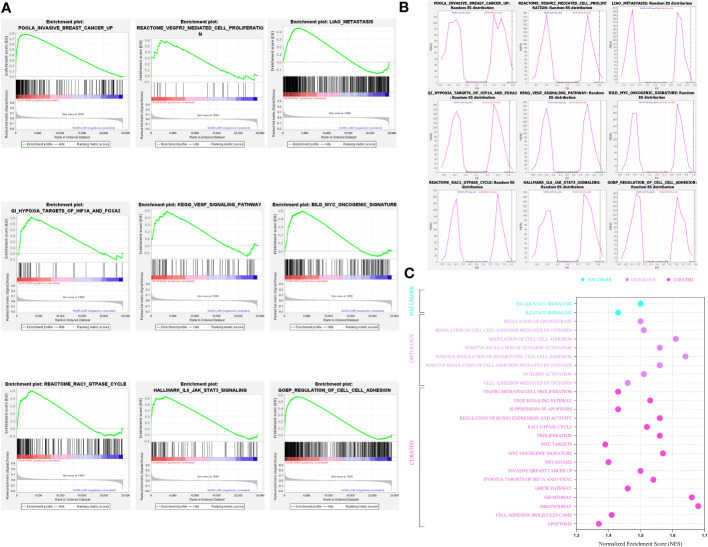
A2AR association with signaling pathways and biological functions involved in breast cancer pathogenesis revealed by Gene Set Enrichment Analysis. **(A)** Gene Set Enrichment Analysis (GSEA) plots illustrate statistically significant and concordant differences in an *a priori* defined set of genes reflecting various biological processes, between A2AR^Low^ and A2AR^High^ clusters. The Plots depict the key pathways implicated in breast cancer development and progression which are positively enriched in A2AR^High^ patients. **(B)** Random ES (Enrichment Score) distribution based on the previous nine enrichment plots. **(C)** The major significant pathways involved in proliferation, invasion, angiogenesis, and metastasis are illustrated in the bubble plot. Hallmark, Ontology and Curated gene sets were exploited as molecular signatures. Enriched terms with a false discovery rate (FDR) and (p-nominal) < 0.05 are considered statistically significant. ES, Enrichment Score; NES, Normalized Enrichment Score.

After shedding light on the clinical and prognostic relevance of A2AR in breast cancer, we attempted to assess its probable involvement in tumor pathogenesis. In this regard, we performed Gene Set Enrichment Analysis (GSEA) to decipher the biological functions and mechanisms implicated in cancer development and progression. According to the Normalized Enrichment Score (NES), analysis of three human molecular signature databases (Hallmark, Curated and Ontology) revealed that the A2AR^high^ phenotype is mainly concentrated in a panoply of gene sets related to oncogenesis and tumor progression (**Figure 8C**). As illustrated in (**Figures 8A, B**), the A2AR is linked to the invasive breast cancer signature, oncogenic and angiogenic signaling pathways (Myc, VEGF and IL6-JAK-STAT3) as well as proliferation, metastasis, hypoxia, adhesion and cell cycle processes (Rac1 GTPASE cycle). In light of these results, A2AR could be a key mediator in the development and progression of human breast cancer.

### 3.5 A2AR^high^ TME exhibits profuse infiltration of protumoral cells and an upregulation of immunosuppressive molecular mediators

**Figure 9:**
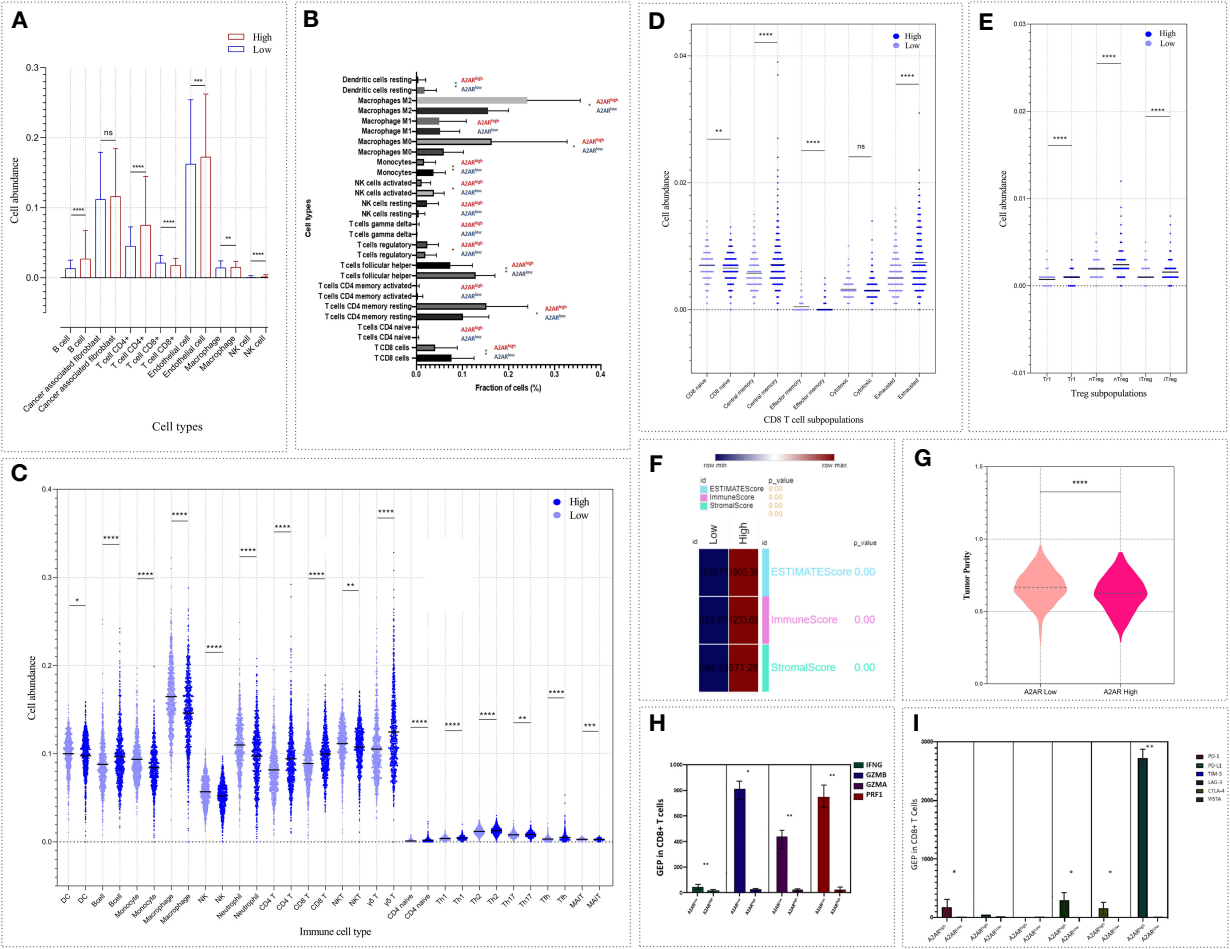
The abundance of cell populations infiltrating the TME reflects an immunosuppressive pattern of A2AR^high^ breast tumors. Four algorithms based on different immune signatures were exploited to analyze the differential distribution of immune cell fractions and tumor purity, ESTIMATE, stromal and immune scores between both groups of patients (A2AR^low^ and A2AR^high^). **(A)** EPIC. **(B)** CIBERSORT. **(C–E)** ImmuneCellAI. **(F, G)** ESTIMATE. **(H)** Bar chart illustrating gene expression of IFNγ, GZMA, GZMB and PRF1 between CD8+ T cells from A2AR^high^ and A2AR^low^ patients. Cells from A2AR^high^ patients show decreased expression of effector and cytotoxic molecules. **(I)** Bar chart depicting the up-regulation of the inhibitory immune checkpoint molecules such as PD-1, LAG-3, CTLA-4 and VISTA on CD8+ T cells from A2AR^high^ patients. Significance was calculated using the Mann-Whitney rank test. *p<0.05, **p<0.01, ***p<0.001, ****p<0.0001, ns, not significant.

In breast cancer, the immune profile of TME plays a critical role in the establishment of patient prognosis and response to immunotherapy. Mellman et al. have provided an overview of the immunologic background for each tumor phenotype. Indeed, tumors exhibiting an immune-inflamed profile testify to a pre-existing immune response marked by upregulation of inhibitory factors and protumoral cell infiltration. Therefore, patients harboring these tumors are more prone to respond to immunotherapy. Since our immunohistochemical analysis revealed an increased prevalence of A2AR in breast tumor infiltrating immune cells, we speculated that A2AR might represent a prominent mediator influencing the composition and abundance of the immune infiltrate. For this purpose, we performed a computational analysis to explore the immune profile of A2AR-related TME, by investigating the composition and abundance of several immune cell subsets in the 1904 patients of METABRIC cohort. To strengthen the validity of our results, the analysis is performed using four different deconvolution algorithms. First, the immune signature of the computational algorithm (EPIC) was used to estimate the proportions of immune and cancer cells (**Figure 9A**). The results show increased infiltration of B cells, CD4+ T cells, NK, macrophages and Endothelial cells within the A2AR^high^ TME. However, CD8+ T cells are significantly more abundant in A2AR^low^ tumors. Subsequently, we used the CIBERSORT (**Figure 9B**) and ImmuneCellAI (**Figures 9C–E**) algorithms to obtain a complete and integrated view of the different cell sub-populations and to identify which cell subsets CD4+, TCD8+, NK, DC and T macrophages infiltrate the A2AR^high^ TME. Interestingly, patients with A2AR^high^ TME displayed profuse infiltration of M0 and M2 macrophages, Treg, Tr1, nTreg, iTreg, T CD4+ memory resting cells, B cells, T γδ, T CD4+ naive, Th1, Th2, Th17, Tfh, Tcm and exhausted T CD8+ cells. However, DC, monocytes, activated NK, NKT, neutrophils, MAIT, effector memory and naive CD8+ T cells appear to be more abundant in A2AR^low^ tumors.

In order to estimate the stromal and immune score and to predict tumor purity, we applied the ESTIMATE enrichment test (**Figures 9F, G**). A2AR^hight^ tumors exhibit high stromal and immune scores. The ESTIMATE score, which represents the non-tumoral component, was also found to be high in this group of patients. Meanwhile, A2AR^high^ TME show lower tumor purity than A2AR^low^ group.

After investigating the cellular components linked to A2AR, we attempted to pinpoint the functional state of CD8+T cells from patients overexpressing this gene (A2AR^high^ CD8+T cells). Expression of effector and cytotoxic molecules (IFNγ, GZMA, GZMB, and PRF1) and inhibitory immune regulators (PD-1, PD-L1, CTLA-4, TIM-3, LAG-3, and VISTA) was assessed. As depicted in (**Figure 9H**), A2AR^high^ CD8+T cells weakly express IFNγ, GZMA, GZMB and PRF1. In contrast, PD-1, CTLA-4, LAG-3, and VISTA exhibit an upregulation in the same group of cells (**Figure 9I**). Therefore, A2AR may also affect the functional state of intratumoral CD8+T cells.

**Figure 10:**
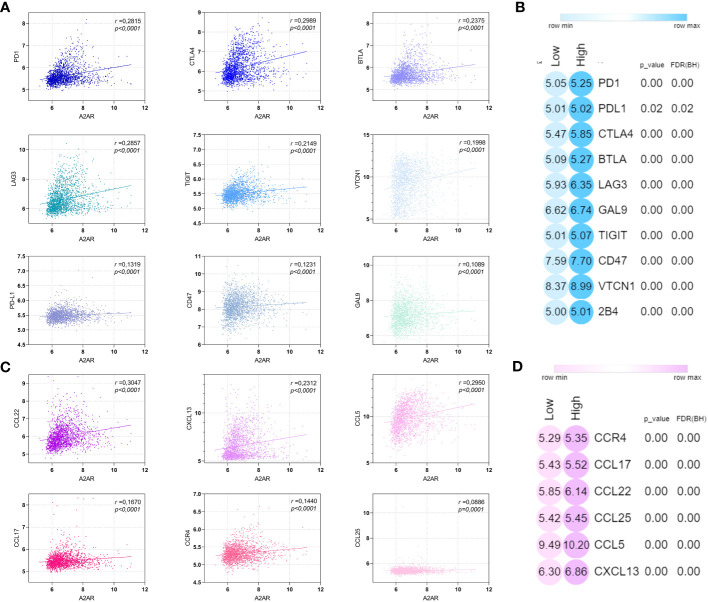
A2AR is positively correlated with immunosuppressive and protumoral molecular mediators. **(A, C)** A2AR exhibits a significant positive correlation with inhibitory immune checkpoint and immunosuppressive chemokines. **(B, D)** Heat maps illustrating the upregulation of inhibitory immune checkpoint and immunosuppressive chemokine in breast cancer patients overexpressing A2AR. Statistical difference was calculated using the Mann-Whitney rank test. Pearson’s rank coefficient was used for correlation.

To further elucidate the relevance of A2AR in TME regulation, we also investigated the pivotal molecular mediators involved in immunosuppression and tumor progression. We therefore assessed the correlation of A2AR with inhibitory immune checkpoints (**Figures 10A, B**) and chemokines (**Figures 10C, D**) involved in the attraction and polarization towards tolerogenic and protumoral cell sub-sets. Thus, A2AR was associated and positively correlated with these immunoregulatory molecules, including the immune checkpoints PD-1, CTLA-4, BTLA, LAG-3, TIGIT, VTCN-1, PD-L1, CD-47 and GAL-9, as well as the chemokines CCL-22, CXCL-13, CCL-5, CCL-17, CCR-4 and CCL-25.

In light of these results, this part of our work illustrates the potential involvement of A2AR in the establishment of the immunosuppressive TME, which is characterized by a pro-tumor cellular component, low tumor purity and an upregulation of major immunosuppressive molecular mediators.

### 3.6 A2AR tends to be prominently expressed on Tregs and exhausted CD8+ T cells

**Figure 11:**
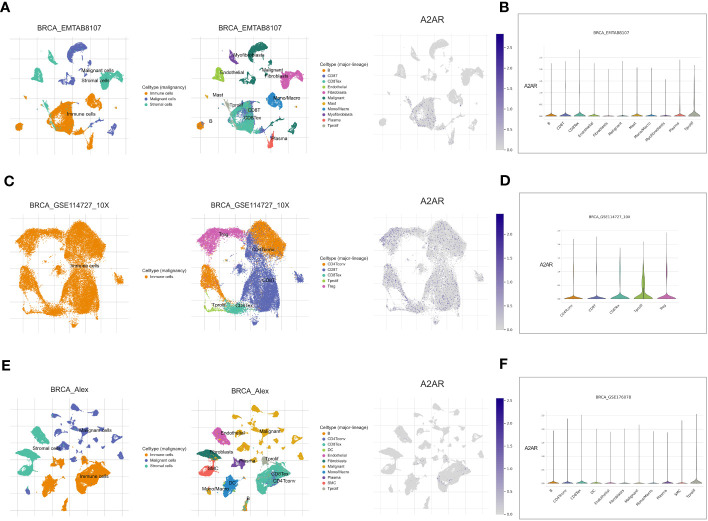
A2AR gene expression in breast TME at single-cell resolution. Analysis is performed using the Tumor Immune Single-cell Hub 2 (TISCH2) scRNA-seq database. Cell type annotation for three datasets: BRCA_EMTAB8107, BRCA_GSE114727_10X, and BRCA_Alex are curated according to two clusters: malignancy and major-lineage. **(A, C, E)** The Uniform Manifold Approximation and Projection (UMAP) dimension reduction learning algorithm was used for interactive visualization of A2AR expression and exploration of cellular landscapes. **(B, D, F)** Violin plot illustrating the distribution of A2AR in different populations of malignant, immune and stromal cells.

To decipher A2AR-expressing cells in the TME, we used the Tumor Immune Single-cell Hub 2 (TISCH2) database. For this purpose, three breast cancer datasets; BRCA_EMTAB8107 (**Figures 11A, B**), BRCA_GSE114727_10X (**Figures 11C, D**) and BRCA_Alex (**Figures 11E, F**), were analyzed. As a first result, A2AR seems to be expressed more in immune cells than in malignant and stromal cells. Subsequently, major lineage data showed that among the different cell populations analyzed, A2AR tends to be prominently expressed on Tregs and exhausted CD8+ T cells. These findings further underscore the potential contribution of A2AR to the immunosuppressive process.

### 3.7 A2AR is involved in immune tolerance and tumor escape processes

**Figure 12:**
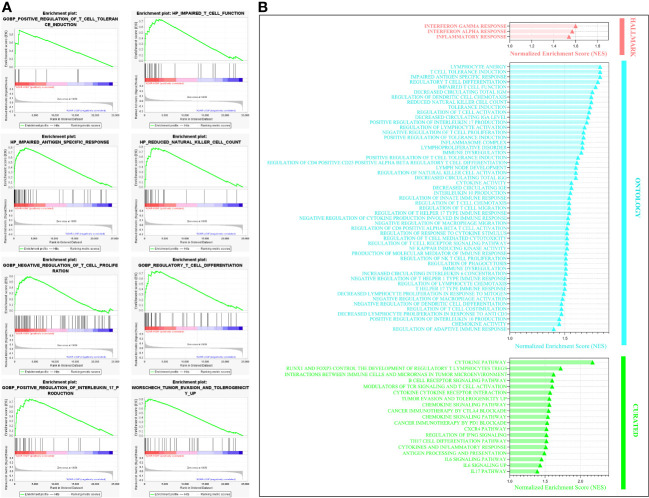
Gene set enrichment analysis illustrating the key immunosuppressive and tumor escape pathways enriched in A2AR^high^ patients. **(A)** Enrichment plot showing eight significant pathways involved in immune tolerance and key cellular effector dysfunction. **(B)** Bar chart of statistically significant immunoregulatory pathways that are positively enriched in A2AR^High^ patients. Hallmark, Ontology and Curated gene sets were exploited as molecular signatures. Enriched terms with a false discovery rate (FDR) and (p-nominal) < 0.05 are considered statistically significant. ES, Enrichment Score, NES, Normalized Enrichment Score.

To further substantiate the protumoral aspect of A2AR^high^ TME, we assessed their immunoregulatory impact using GSEA enrichment analysis. As illustrated in (**Figure 12B**), a wide range of immunosuppression and tumor escape-related gene-sets is positively enriched in A2AR^high^ TME. These pathways mainly involve the dysfunction and downregulation of T cell proliferation, impaired antigen-specific response, reduced natural killer cell count, upregulation of IL-17 production, tumor escape and tolerogenicity (**Figure 12A**, **Supplementary Figure 1**).

Therefore we can suggest that A2AR represents a potent immunosuppression mediator and a promising target for immunotherapy to overcome the immune evasion prevalent in human breast cancer.

## 4 Discussion

The TME reflects a dynamic network wherein tumor and immune cells interplay is strictly mediated by molecular effectors promoting tumor progression (61, 62). The main constraint for breast cancer to elicit an effective antitumor response resides in its highly immunosuppressive profile. Immune evasion constitutes a critical step in breast tumor progression, where inhibitory immune checkpoint molecules represent a crucial protumoral mediator (63, 64). Thus, to overcome and defeat immune escape, the ICIs targeting PD-1 and CTLA-4 have been conceived as an emerging immunotherapeutic strategy. This treatment approach has proven promising, however, efficient and long-lasting responses occur among a restricted group of patients (65). In this regard, Atezolizumab (anti-PD-L1), the only FDA-approved immunotherapeutic agent for breast cancer is unfortunately limited to metastatic TNBC (66). The unresponsiveness to current ICIs could be ascribed to the post-therapeutic upregulation of other compensatory immune checkpoints such as A2AR (56, 67, 68). This mechanism is often adopted by tumors to counterbalance and offset the immunosuppressive effect of the blocked molecule (69). Furthermore, one third of invasive breast cancers exhibit hypoxic TME, which could promote the HIF-1α-A2A-adenosinergic pathway, and consequently the establishment of immunosuppression (70, 71). All these facts sparked our interest in bringing to light the clinical and prognostic relevance of A2AR and its related immunological profile in breast cancer. Accordingly, the first part of this work focused on transcriptomic and proteomic analysis in two distinct breast cancer cohorts. Our experimental study revealed that breast tumors exhibited increased levels of A2AR transcript compared to uninvaded control tissues. This overexpression was related to high grade, ER- and PR- status as well as HER2+ and TNBC molecular subtypes. Protein analysis has supported the transcript level results with an additional association to the Ki-67 proliferation index. Nevertheless, this observation was noted exclusively in immune cells, hinting that A2AR severely affects patient clinical prognosis probably via the immune axis regulation. These findings were confirmed by METABRIC cohort, wherein A2AR expression was associated with high grade, aggressive histological subtypes, as well as PR- and HER2+ status. Interestingly, in addition to HER2+ molecular subtype, a strong expression of this inhibitory receptor was observed in Claudin-low tumors. The latter represents a group of patients who manifest poor survival (59). Moreover, the Nottingham Prognostic Index reported that patients predicting short 10-year survival displayed high levels of A2AR. Kaplan-Meier analysis further demonstrated the prognostic significance of A2AR by showing its association with worse survival in breast cancer patients. In gastric and colorectal cancers, A2AR protein appears to be overexpressed with a correlation to disease progression and reduced survival (48, 53). Head and neck squamous cell carcinoma samples also showed elevated expression of this protein, which was linked to advanced pathologic grade, larger tumor size, positive lymph node status, recurrence, and poor survival (47). Similar results were observed in renal cell carcinoma where A2AR was associated with metastatic profiles. It was also found that patients with A2AR^high^ status did not respond efficiently to anti-VEGF or anti-PD-1 monotherapy as well as to combined therapy with anti-PD-1 and anti-CTLA-4 (52). In agreement with our findings, all these observations testify to the aggressive clinical outcomes and poor prognosis of A2AR elevated expression in cancer.

Although ICIs monotherapy has emerged as an appealing strategy, the synergistic effect of multi-targeted blockade has brought considerably superior benefits (39, 67, 72–74). In fact, the relevance of combined therapy mirrors the cooperative interaction between negative regulators, which simultaneously collaborate to achieve immune tolerance (26, 52, 73, 75). Co-inhibition of A2AR and PD-1 or CTLA-4 has been investigated in several types of cancer and proven promising for the clinical application (39, 67, 72). However, the potential interplay between A2AR and PD-1 or CTLA-4 has not yet been elucidated in human breast cancer. In this regard, we have explored the correlation between A2AR and these two inhibitory receptors in the mammary TME. As a first observation, compared to PD-1 and CTLA-4, A2AR appears as the most highly expressed protein in breast cancer tumors. This could imply that the immunosuppression occurring in breast TME might be further orchestrated by A2AR pathway. As expected, our experimental results also revealed the positive correlation between A2AR and PD-1. Therefore, we can speculate that inherent interdependence may exist between these two receptors to synergistically amplify immune escape. Compared to single agent treatment, dual blockade of A2AR and PD-1 pathways exhibited a significant improvement in immune response restoration, tumor growth inhibition and survival in preclinical models of breast and colorectal cancer (39, 67, 74, 76). In metastatic renal cell carcinoma patients treated with anti-PD-1, increased A2AR expression was associated with poor treatment response and reduced survival (52). Accordingly, the phase 1/1b clinical trials conducted on refractory renal and non-small cell lung cancer patients reported that A2AR antagonism showed antitumor activity with clinical responses, even in patients resistant or refractory to prior anti-PD-1/PD-L1 treatment (56, 77). Otherwise, CD73/A2AR and PD-1/PD-L1 signaling was found to induce immunosuppressive TME in diffuse large B-cell lymphoma (78). Indeed, patients whose CD8+T cells co-express both A2AR and PD-1 had shorter overall and progression-free survival than those whose CD8+T cells solely express either A2AR or PD-1 (75). Furthermore, studies have shown that A2AR stimulation would impact the regulation of PD-1/PD-L1 pathway, thereby supporting the interactive relationship between these two immune checkpoints. As a matter of fact, A2AR activation upregulates PD-1 on tumor-specific CD8+T and Treg cells, whereas its inhibition decreases the expression of PD-L1 on myeloid APCs and PD-1 on both tumor-associated CD8+T and Tregs cells (74, 79, 80).

In turn, concomitant blockade of A2AR and CTLA-4 also proved quite beneficial in various experimental models. A2AR antagonism was proven to significantly enhance the antitumor activity of anti-CTLA-4 in colorectal, renal, melanoma, prostate and metastatic breast cancer models (39, 72–74). It has been reported that co-targeting these two immunosuppressive pathways exhibited improved immune response with prolonged survival, whereas monotherapy showed partial efficacy (39, 72, 73). We therefore investigated the correlation between A2AR and CTLA-4 expression in our breast cancer patients. Surprisingly, in contrast to PD-1, we found that A2AR is negatively correlated with CTLA-4. Indeed, many studies have revealed that down-regulation of immune checkpoint molecules could induce the compensatory expression and stimulation of other immunosuppressive pathways. PD-1 deficient mice were found to overexpress the CTLA-4 protein (26, 81). Meanwhile, inhibition of CTLA-4 also results in upregulation of PD-1 and adenosinergic genes (72, 81). Consequently, we can suggest that the cooperative mechanism of immune checkpoints does not always rely on concomitant action, but also on compensatory feedback loops.

The composition of tumor-infiltrating immune cells is of major prognostic relevance, given its key role in disease growth and development as well as response to treatment. The TME harbors different cell types, which can either favor tumor progression or conversely serve an antitumor function (62, 82). ESTIMATE, stromal and immune score computation revealed low tumor purity and abundant stromal and immune infiltration in A2AR^high^ tumors. In fact, low tumor purity is an independent poor prognostic factor. Previous studies have shown the significant association of this tumor feature with short survival, early relapse, invasive and metastatic phenotype, EMT, upregulation of inhibitory immune checkpoints and immunosuppressive chemokines as well as high infiltration of protumoral cells, including M2 macrophages and Tregs (83, 84).

Subsequently, investigating the profile of tumor-infiltrating cell, we found that compared to the A2AR^low^ phenotype, TME with a strong A2AR expression had an increased proportion of protumoral cells, including M0 and M2 macrophages, different subsets of Tregs (Tr1, nTreg and iTreg), exhausted T CD8+ cells and CD4+ memory resting T cells. The association between M0 macrophages and unfavorable patient prognosis has been illustrated in several tumor contexts. In breast cancer, a high fraction of this cell subset correlates positively with high grade, high Ki-67 proliferative index and poor overall and disease-free survival (85–89). Whereas the M2 phenotype has been shown to have proangiogenic activity promoting breast cancer metastasis and to be closely related to worse clinical outcomes (87, 89, 90). The polarization of monocytes into tolerogenic M2-like macrophages known for their weak proinflammatory effect could occur in response to A2AR stimulation. The protumoral behavior of this cell type lies in its high expression of IL-10, arginase 1, iNOS and VEGF with low expression of TNF and IL-12 cytokines (45, 91).

In turn, the frequency of Treg cells represents a useful hallmark for breast cancer prognosis. A higher fraction of Foxp3+ Tregs correlates positively with ER-, PR- and HER2+ status, nodal invasion and short survival (92, 93). However, the decrease in Treg abundance was associated with the complete pathological response in TNBC patients who underwent adjuvant chemotherapy (94). Taylor et al. reported that Tregs exhibit a substantial proportion of Claudin-low tumor-infiltrating lymphocytes. They have also shown that Tregs isolated from Claudin-low tumor-bearing mice display a strongly immunosuppressive function capable of inhibiting T cell proliferation and effector response (95). The activation of A2AR increases the intracellular rate of cAMP and HIF-1α in Tregs, which triggers the downstream signal transduction cascades leading to enhanced transcription of genes involved in Tregs development and function including; Foxp3, IL-10, TGFβ, GAL-1, PD-1, CTLA-4 and LAG-3 (46, 96–100). A2AR+Tregs are able to establish an immunosuppressed state of TME by upregulating CD39 and CD73 ectoenzymes, resulting in eADO release, which in turn induces inhibition of Teff lymphocytes (40, 46, 47, 97, 99). This eADO can also operate in an autocrine loop by feeding back to Tregs the transducing stimulus of rising intracellular cAMP via its A2AR receptor (97, 100). These observations were crowned by works of pharmacological blockade and gene silencing of A2AR in experimental models, highlighting the immunosuppressive impact of this receptor when expressed on Tregs (40, 47, 100).

Meanwhile, substantial abundance of CD4+ memory resting T cells is associated with unfavorable prognosis in gastric cancer (101). Nevertheless, prolonged survival and remarkable response to ICIs as well as increased tumor mutational burden and neoantigen load were observed in melanoma patients with a profuse infiltration of CD4+ memory activated T cells and a lower fraction of CD4+ memory resting T cells (102).

It is noteworthy that cell infiltrate analysis also portrays a reduced proportion of cells mediating antitumor activity, notably DC, activated NK, NKT and effector memory CD8+ T cells in A2AR^high^ patients. It is clearly established that the presence of the above-mentioned cells within breast TME correlates positively with prolonged survival, prevention of metastatic progression and complete pathological response, consequently affording better prognosis for patients (103–109).

In NK cells, A2AR is regarded as an intrinsic negative regulator of the maturation and effective killing function of this cell type. Targeting this ADO-receptor results in reduced metastasis, improved tumor control and delayed tumor initiation in experimental models, by enhancing NK-mediated cytotoxic activity in a PRF1 and GZMB-dependent manner (42, 110). Furthermore, during infection and cancer, A2AR engagement seems to inhibit via IL-15 signaling blockade, the generation of human CD39+NK cells endowed with a potent degranulation capacity and overexpression of IFNγ and TNFα (111).

Several works have provided through *in vitro* systems and various murine models a clear evidence of A2AR-mediated CD8+T cell exhaustion (39–41, 68, 112). By impairing upstream TCR signaling, A2AR downregulates NOTCH1 pathway, leading to reduced production of IFNγ, PRF1 and GZMB (39–41). Moreover, restricted CD8+T cell proliferative potential has been described in A2AR-deficient mice (36). In this regard, our study aimed to investigate the expression impact of this ADO-receptor on the functional state of human breast tumor-infiltrating CD8+ T cells. Our digital cytometry analysis revealed a very weak expression of effector and cytotoxic molecules, including IFNγ, GZMA, GZMB and PRF1 within CD8+T cells from A2AR^high^ patients. In contrast, an upregulation of negative regulators such as PD-1, CTLA-4, LAG-3 and VISTA was observed within this cell cluster. The inhibitory immune checkpoints included in the analysis are well established markers of CD8+T cell depletion (113–116). Based on these observations, our results provide some evidence of the impact of A2AR on the dysfunctional profile of CD8+T cells in breast cancer. Interestingly, Single-cell data corroborate these findings, showing that A2AR tends to be upregulated on exhausted CD8+ T cells and Tregs. As a matter of fact, recent study repoted that pharmacological and genetic targeting of A2AR substantially enhanced the clinical efficacy of CAR-T-cell therapy by promoting their activation, effector cytokine production and antitumor activity in breast tumor-bearing mice (68). A2AR antagonism has also improved melanoma patient-derived CAR-T-cell activity (68).

Admittedly, the cellular component has a major impact on cancer prognosis. However, molecular factors released by immunosuppressive TME cells and/or promoting their attraction and polarization towards a protumoral and tolerogenic phenotype play a pivotal role and reflect the aggressive tumor behavior. We therefore studied the association of our gene of interest with a panel of inhibitory immune checkpoints, including PD-1, CTLA-4, BTLA, LAG-3, TIGIT, VTCN-1, PD-L1, CD-47 and GAL-9, as well as immunosuppressive chemokines such as CCL-22, CXCL-13, CCL-5, CCL-17, CCR-4 and CCL-25. Thus, A2AR was found to be positively correlated with these well-known mediators of immune evasion.

Finally, the last part of our work focused on enrichment analysis to provide further evidence for A2AR involvement in breast cancer pathogenesis. Thus, the present study revealed the close association of this inhibitory immune checkpoint with the invasive breast cancer signature as well as the mechanisms of immunosuppression, tumor escape, proliferation, hypoxia, angiogenesis and metastasis. In the light of these findings and to the best of our knowledge, this work is the first to elucidate the clinical and immunological relevance of A2AR in breast cancer. Considering its link to dismal clinical outcomes and unfavorable prognosis, we have provided compelling evidence for the involvement of this ADO-receptor in the aggressiveness of the disease. Furthermore, the present study underlines the link between A2AR and the mechanisms of immunosuppression and tumor development and progression.

Despite significant advances in the management of breast cancer, it remains a major public health problem. Although immunotherapy with current immune checkpoint inhibitors has attracted a great deal of interest, they remain ineffective in breast cancer. It is necessary to explore new potential biomarkers to improve patient prognosis. Accordingly, our work suggests that A2AR could be considered a promising therapeutic target for human breast cancer. Moreover, its use as part of a combination therapy might enhance the efficacy of currently available ICIs.

## Data availability statement

The original contributions presented in the study are included in the article/**Supplementary Materials**, further inquiries can be directed to the corresponding author/s.

## Ethics statement

The studies involving humans were approved by the Ethics Committee for Biomedical Research (CERB) of Ibn Rochd University Hospital Center, under the approval code (28/15). The studies were conducted in accordance with the local legislation and institutional requirements. The participants provided their written informed consent to participate in this study.

## Author contributions

Study conception and design: BZ and AB. Data acquisition: BZ, IRe, ME, and MK. Technical support: DC, IRe, HB, and DO. Data analysis: BZ, DC, and AB. Data interpretation: BZ, IRa, MK, and AB. Manuscript drafting and editing: BZ and AB. All authors contributed to the article and approved the submitted version.

## Supplementary material

Supplementary Figure 1 Random (ES) distribution plot of eight statistically significant pathways involved in immunosuppression and tumor evasion. Gene sets related to immune tolerance and immune cell dysfunction are enriched in the A2AR^High^ group. Hallmark, Ontology and Curated gene sets were exploited as molecular signatures. Enriched terms with a false discovery rate (FDR) and (p-nominal) < 0.05 are considered statistically significant. ES: Enrichment Score.
