# Supplementary material for: The immune checkpoint adenosine 2A receptor is associated with aggressive clinical outcomes and reflects an immunosuppressive tumor microenvironment in human breast cancer
Source: Front Immunol. 2023 Sep 11;14:1201632. doi: 10.3389/fimmu.2023.1201632 (PMC10518422; doi:10.3389/fimmu.2023.1201632)
Supplement: Supplementary file 2 [file Table_1.docx]

**Supplementary Table S1. Clinicopathological parameters of the METABRIC cohort**

| Clinicopathological parameters | No. | (%) |
| --- | --- | --- |
| Histological subtypes  Mucinous  Ductal  Lobular  Mixed | 22  1500  142  207 | 1.18  80.17  7.59  11.06 |
| Histological grade  Grade I  Grade II  Grade III | 165  740  927 | 9.00  40.40  50.60 |
| Nottingham prognostic index (NPI)  Excellent  Good  Moderate  Poor | 163  477  1070  194 | 8.56  25.05  56.20  10.19 |
| Molecular subtypes  Normal  Luminal A  Luminal B  HER2+  Basal  Claudin-Low | 140  679  461  220  199  199 | 7.38  35.77  24.29  11.60  10.48  10.48 |
| Estrogen receptor status (ER)  ER+  ER- | 1459  445 | 76.63  23.37 |
| Progesterone receptor status (PR)  PR+  PR- | 1009  895 | 53.00  47.00 |
| HER2 status  HER2-  HER2+ | 1668  236 | 87.60  12.40 |
| Ki-67 proliferation index  Ki-67 Low  Ki-67 High | 619  603 | 50.65  49.35 |

HER-2: human epidermal growth factor receptor-2, TNBC: triple negative breast cancer, ER: estrogen receptor and PR: progesterone receptor.
